# Aberrant intestinal microbiota in individuals with prediabetes

**DOI:** 10.1007/s00125-018-4550-1

**Published:** 2018-01-29

**Authors:** Kristine H. Allin, Valentina Tremaroli, Robert Caesar, Benjamin A. H. Jensen, Mads T. F. Damgaard, Martin I. Bahl, Tine R. Licht, Tue H. Hansen, Trine Nielsen, Thomas M. Dantoft, Allan Linneberg, Torben Jørgensen, Henrik Vestergaard, Karsten Kristiansen, Paul W. Franks, the IMI-DIRECT consortium, Torben Hansen, Fredrik Bäckhed, Oluf Pedersen

**Affiliations:** 1 Novo Nordisk Foundation Center for Basic Metabolic Research, Section for Metabolic Genetics, Faculty of Health and Medical Sciences, University of Copenhagen, Blegdamsvej 3B, DK-2200 Copenhagen, Denmark; 2 Department of Clinical Epidemiology, Bispebjerg and Frederiksberg Hospital, the Capital Region, Copenhagen, Denmark; 3 Wallenberg Laboratory, Department of Molecular and Clinical Medicine, University of Gothenburg, SE-413 45 Gothenburg, Sweden; 4 Sahlgrenska Center for Cardiovascular and Metabolic Research, University of Gothenburg, Gothenburg, Sweden; 5 Laboratory of Genomics and Molecular Biomedicine, Department of Biology, Faculty of Science, University of Copenhagen, Copenhagen, Denmark; 6 National Food Institute, Technical University of Denmark, Lyngby, Denmark; 7 Research Centre for Prevention and Health, the Capital Region of Denmark, Copenhagen, Denmark; 8 Department of Clinical Experimental Research, Rigshospitalet, Glostrup, Denmark; 9 Department of Public Health, Faculty of Health and Medical Sciences, University of Copenhagen, Copenhagen, Denmark; 10 Faculty of Medicine, Aalborg University, Aalborg, Denmark; 11 Steno Diabetes Center Copenhagen, Gentofte, Denmark; 12 Department of Clinical Sciences, Genetic and Molecular Epidemiology Unit, Lund University, Malmö, Sweden; 13 Department of Public Health and Clinical Medicine, Umeå University, Umeå, Sweden; 14 Department of Nutrition, Harvard T.H. Chan School of Public Health, Boston, MA USA; 15 Faculty of Health Sciences, University of Southern Denmark, Odense, Denmark; 16 Novo Nordisk Foundation Center for Basic Metabolic Research, Section for Metabolic Receptology and Enteroendocrinology, Faculty of Health and Medical Sciences, University of Copenhagen, Copenhagen, Denmark; 17 Faculty of Health Sciences, University of Aarhus, Aarhus, Denmark

**Keywords:** *Akkermansia muciniphila*, *Clostridium*, Faecal transfer, Gut microbiota, Hyperglycaemia, Intestinal microbiota, Low-grade inflammation, Prediabetes

## Abstract

**Aims/hypothesis:**

Individuals with type 2 diabetes have aberrant intestinal microbiota. However, recent studies suggest that metformin alters the composition and functional potential of gut microbiota, thereby interfering with the diabetes-related microbial signatures. We tested whether specific gut microbiota profiles are associated with prediabetes (defined as fasting plasma glucose of 6.1–7.0 mmol/l or HbA_1c_ of 42–48 mmol/mol [6.0–6.5%]) and a range of clinical biomarkers of poor metabolic health.

**Methods:**

In the present case–control study, we analysed the gut microbiota of 134 Danish adults with prediabetes, overweight, insulin resistance, dyslipidaemia and low-grade inflammation and 134 age- and sex-matched individuals with normal glucose regulation.

**Results:**

We found that five bacterial genera and 36 operational taxonomic units (OTUs) were differentially abundant between individuals with prediabetes and those with normal glucose regulation. At the genus level, the abundance of *Clostridium* was decreased (mean log_2_ fold change −0.64 (SEM 0.23), *p*_*adj*_ = 0.0497), whereas the abundances of *Dorea*, [*Ruminococcus*], *Sutterella* and *Streptococcus* were increased (mean log_2_ fold change 0.51 (SEM 0.12), *p*_*adj*_ = 5 × 10^−4^; 0.51 (SEM 0.11), *p*_*adj*_ = 1 × 10^−4^; 0.60 (SEM 0.21), *p*_*adj*_ = 0.0497; and 0.92 (SEM 0.21), *p*_*adj*_ = 4 × 10^−4^, respectively). The two OTUs that differed the most were a member of the order Clostridiales (OTU 146564) and *Akkermansia muciniphila*, which both displayed lower abundance among individuals with prediabetes (mean log_2_ fold change −1.74 (SEM 0.41), *p*_*adj*_ = 2 × 10^−3^ and −1.65 (SEM 0.34), *p*_*adj*_ = 4 × 10^−4^, respectively). Faecal transfer from donors with prediabetes or screen-detected, drug-naive type 2 diabetes to germfree Swiss Webster or conventional C57BL/6 J mice did not induce impaired glucose regulation in recipient mice.

**Conclusions/interpretation:**

Collectively, our data show that individuals with prediabetes have aberrant intestinal microbiota characterised by a decreased abundance of the genus *Clostridium* and the mucin-degrading bacterium *A. muciniphila*. Our findings are comparable to observations in overt chronic diseases characterised by low-grade inflammation.

**Electronic supplementary material:**

The online version of this article (10.1007/s00125-018-4550-1) contains peer-reviewed but unedited supplementary material, which is available to authorised users.


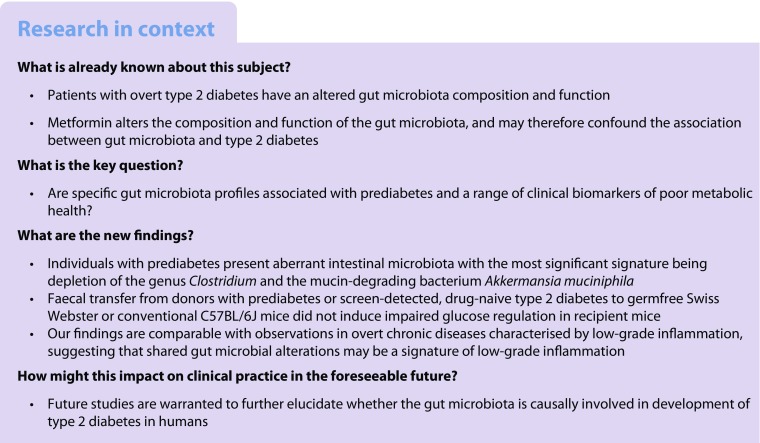


## Introduction

The pathogenesis of type 2 diabetes is a continuous process, during which blood glucose levels gradually rise due to increasing insulin resistance and decreasing beta cell function [1]. Prediabetes is defined by blood glucose levels that are higher than normal but lower than the thresholds set for diagnosing diabetes. Individuals with prediabetes often present with overweight, insulin resistance and low-grade inflammation, and they have an increased risk of type 2 diabetes and ischaemic cardiovascular disease [2].

Several studies have suggested that altered gut microbiota composition and function are associated with overt type 2 diabetes [3–7] and atherosclerosis [8]. However, in a recent study, we called into question previously reported associations between aberrant gut microbiota and type 2 diabetes by demonstrating that metformin, the first choice drug for treatment of hyperglycaemia in type 2 diabetes, confounds this relationship [9]. Accordingly, although gut microbial signatures can be used with high accuracy to distinguish individuals with type 2 diabetes who are not stratified by mode of treatment from healthy individuals, this is not the case when attempted in metformin-naive individuals with type 2 diabetes [9]. An alternative approach to post hoc stratification to circumvent confounding by drug treatment is to study individuals with prediabetes who are not currently treated with glucose-lowering drugs.

Therefore, the aim of the present study, which included 268 Danish individuals with prediabetes or normal glucose regulation, was to test the hypothesis that specific gut microbiota profiles are associated with prediabetes and a range of clinical biomarkers of poor metabolic health. We also tested the hypothesis that the intestinal microbiota directly modulate host glucose metabolism by transferring faecal microbiota from human donors to germfree Swiss Webster and conventional C57BL/6 J mice.

## Methods

### Study population

We included 134 individuals with prediabetes and 134 individuals with normal glucose regulation. In accordance with the criteria from the WHO [10, 11], prediabetes was defined as fasting plasma glucose levels of 6.1–7.0 mmol/l or HbA_1c_ levels of 42–48 mmol/mol (6.0–6.5%). Normal glucose regulation was defined according to the more strict ADA criteria [12], i.e. fasting plasma glucose <5.6 mmol/l and HbA_1c_ <39 mmol/mol (5.7%). Individuals with known diabetes and individuals receiving any glucose-lowering drugs were ineligible for inclusion. Individuals with prediabetes were recruited from the Danish part of the Innovative Medicines Initiative – DIabetes REseraCh on patient straTification (IMI-DIRECT) (*n* = 63) and the Danish study of Functional Disorders (DanFunD) (*n* = 71) [13, 14]. At the initiation of the present study, 228 Danish individuals were eligible from IMI-DIRECT and 598 were eligible from DanFunD; all individuals fulfilling the abovementioned inclusion and exclusion criteria were included. Individuals with normal glucose regulation were recruited only from DanFunD and matched 1:1 to individuals with prediabetes with respect to sex and age (*n* = 134). Dietary habits could only be reliably compared between individuals with prediabetes and normal glucose regulation in a subset of the cohort (*n* = 142) where dietary information was obtained by identical questionnaires.

All participants gave written informed consent and both studies conformed to the principles of the Declaration of Helsinki and were approved by the Regional Scientific Ethical Committee, the Capital Region of Denmark (H-1-2011-166 and H-3-2012-015).

### Biochemical analyses and anthropometrics

Biochemical analyses were performed on fasting blood samples. Plasma insulin and C-peptide were measured by chemiluminometric immunoassays (DiaSorin Liaison Analyzer, DiaSorin, Saluggia, Italy, at University of Eastern Finland, Kuopio, Finland). Plasma high-sensitivity C-reactive protein (hsCRP) was measured by an immunoturbidimetry assay (Roche/Hitachi analyser, Roche, Basel, Switzerland, at Vejle Hospital, Vejle, Denmark). HbA_1c_, plasma glucose and triacylglycerol were measured at the Steno Diabetes Center, Gentofte, Denmark (IMI-DIRECT participants) or Rigshospitalet Glostrup, Copenhagen University Hospital, Denmark (DanFunD participants). HbA_1c_ was measured by high-performance liquid chromatography (TOSOH analyser, TOSOH, Tokyo, Japan) and plasma triacylglycerol by colorimetric slide analysis (Vitros analyser, Ortho Clinical Diagnostics, Raritan, NJ, USA). In IMI-DIRECT, plasma glucose was measured twice by a glucose hexokinase assay (Konelab Analyser, Thermo Fisher Scientific, Waltham, MA, USA) and the mean was calculated. In DanFunD, plasma glucose was measured by colorimetric slide analysis (Vitros analyser). Insulin resistance was estimated as HOMA-IR: (fasting plasma glucose [mmol/l] × fasting serum insulin [pmol/l])/135.

Body weight (kg) and height (cm) were measured in light indoor clothes and without shoes. Waist circumference was measured midway between the iliac crest and the lower costal margin.

### Collection of faecal samples and extraction of faecal genomic DNA

Fresh stool samples were collected by the participants at home and were immediately frozen in their home freezer at about −20°C. Frozen samples were transported to the laboratory using insulating polystyrene foam containers or dry ice. The maximum transportation time in the insulating polystyrene foam containers was 4 h and at delivery study nurses ensured that all samples were frozen. After delivery, the samples were stored at −80°C until DNA extraction. Total faecal genomic DNA was extracted from 300 mg of faecal material using the NucleoSpin Soil kit (Macherey-Nagel, Düren, Germany) [15]. Briefly, the faecal material was suspended in SL2 buffer containing SX enhancer and cell disruption was carried out by bead beating at 30 Hz for 5 min using a TissueLyser instrument (Qiagen, Hilden, Germany).

### Profiling of faecal microbiota composition by sequencing of the 16S rRNA gene

Faecal microbiota composition was profiled by sequencing the V4 region of the 16S rRNA gene on an Illumina MiSeq instrument (llumina RTA v1.17.28; MCS v2.5) with 515F and 806R primers designed for dual indexing [16] and the V2 Illumina kit (2 × 250 bp paired-end reads). Details on amplification of 16S rRNA genes are provided in the electronic supplementary material (ESM) Methods.

Illumina reads were merged using PEAR [17] and quality filtered by removing all reads that had at least one base with a q-score <20. Quality filtered reads were analysed with the software package QIIME, version 1.8.0 (http://qiime.org) [18] as described in ESM Methods. Chimeric sequences, low abundant operational taxonomic units (OTUs; relative abundance <0.002%) and OTUs that could not be aligned with PyNAST [19] were excluded from all analyses. We obtained a mean ± SD of 48,169.6 ± 11,131.7 sequences/sample (range 26,968–87,208). In total, 12,909,447 sequences and 1609 OTUs were included in the analyses. To correct for differences in sequencing depth between samples, 26,968 sequences were randomly subsampled from each sample and included in the analyses for the estimation of α- and β-diversity. OTUs that showed differential abundance among individuals with prediabetes and normal glucose regulation were blasted against the NCBI 16S ribosomal RNA sequences (Bacteria and Archaea) database to obtain a more specific annotation.

### Transfer of gut microbiota to conventional mice

From the study population of 268 individuals, we selected four individuals with the poorest (fasting plasma glucose >6.1 mmol/l and HbA_1c_ >42 mmol/mol [6.0%]) and four individuals with the best glucose regulation (lowest levels of fasting plasma glucose). Case–controls were matched with respect to sex. Frozen stools (250 mg) were obtained from each donor. Case stools and control stools were pooled separately and re-suspended in 4.5 ml PBS prior to each transfer. Mice (48 male wild-type C57BL/6 J, Taconic, Lille Skensved, Denmark; 10 weeks of age) were co-housed with three mice per cage and kept at 22°C under a 12 h light cycle and fed ad libitum with free access to water. After 2 weeks of acclimatisation on a standard chow diet (Altromin 1310, Altromin, Lage, Germany), mice were transferred to a Western diet (D12079 mod.* customised; sucrose was the main carbohydrate source with mixed protein and fat sources) and sham-gavaged with PBS twice a week. After 3 weeks on the Western diet, mice were divided into two equal recipient groups of 18 mice each (six cages per group) and one sham-gavaged control group of 12 mice (four cages per group). The groups were stratified based on magnetic resonance determined fat mass, weight and response to a glucose challenge prior to intervention. For the following 4 weeks, mice were gavaged three times per week with 200 μl of either faecal microbiota (control or case) or PBS (sham) while remaining on the Western diet. Two mice (one case and one control) died during the experiment and were excluded from all analyses.

An OGTT was performed after 4 weeks of gavaging. The mice were fasted for 5 h and gavaged with 3 g glucose/kg lean mass. Blood glucose was measured in tail vein blood at 0, 15, 30, 60, 90 and 120 min using standard Contour Next Test Strips (Bayer Contour, Leverkusen, Germany). Plasma insulin was measured before the glucose bolus and during the OGTT using an electrochemiluminescence assay (Meso Scale Diagnostics, Rockville, MD, USA). Colonisation of the recipient mice by the human gut microbiota was tested by extraction of total genomic DNA from faecal samples collected before and at the end of the 4 week gavage period, as well as caecal samples at the end of the experiment, followed by sequencing of the 16S rRNA gene as described above. Faecal and caecal samples were randomised before DNA extraction.

All animal experiments were conducted in accordance with national Danish guidelines (Amendment number 1306 of 23 November 2007) as approved by the Danish Animal Inspectorate, Ministry of Justice, permission number 2014-15-2934-01027. Mice were kept under specific-pathogen-free conditions and experimental protocols were validated by in-house standard operation procedures.

### Transfer of gut microbiota to germfree mice

From the IMI-DIRECT study, we selected two male donor pairs each consisting of one individual with screen-detected type 2 diabetes (according to WHO criteria) and one with normal glucose regulation (according to ADA criteria). Germfree Swiss Webster mice were bred in-house. Five to six 10-week-old male Swiss Webster mice fed regular chow diet were transplanted with faeces from each donor. Mice were kept in individually ventilated cages (ISOcage N System, Tecniplast, Buguggiate, Italy) with a maximum of five mice per cage under a 12 h light cycle and a room temperature of 21°C. Water was given ad libitum. Frozen stools (500 mg) obtained from each human donor were suspended in 5 ml of reduced PBS. The mice were divided into two groups based on body weight and colonised by oral gavage with 200 μl of faecal slurry from each donor. Two weeks after colonisation an intraperitoneal GTT was performed. Mice were fasted for 4 h and injected with d-glucose (2 g/kg body weight). Blood glucose was measured in tail vein blood at 0, 15, 30, 60, 90 and 120 min with a Contour Next EZ glucometer (Bayer, Leverkusen, Germany). Additional blood samples were collected at 0, 15 and 30 min to analyse plasma insulin levels by insulin ELISA Crystal Chem (Downers Grove, IL, USA). Colonisation of the recipient mice by the human gut microbiota was examined in caecal samples collected at the end of the experiment as described above. Caecal samples were randomised before DNA extraction.

All animal procedures were approved by the Gothenburg Animal Ethics Committee (152-2015).

### Statistical analyses

Statistical analyses were performed using R version 3.2.1 (www.r-project.org) and Stata, version 13.1 (StataCorp, College Station, TX, USA). Differences in baseline characteristics between individuals with prediabetes and those with normal glucose regulation were assessed by use of Wilcoxon rank ∑ tests for continuous variables and *χ*^2^ tests for categorical variables. Differences in microbiota composition as assessed by β-diversity metrics were tested using multivariate non-parametric ANOVA [20] implemented in the Adonis function (999 permutations) in the R vegan package [21]. Differences in α-diversity were tested using a two-sample unpaired *t* test and associations between α-diversity and clinical biomarkers were tested using simple linear regression analyses. Only taxa present in at least 50% of the samples were included in the analysis of differential abundance. Differences in abundance of genera and OTUs were tested using a negative binomial wald test implemented in the R DESeq2 package [22, 23]. When examining differential abundance of genera and OTUs, *p* values were adjusted (*p*_*adj*_) for multiple testing by the Benjamini–Hochberg procedure. Correlation between taxa abundances and clinical biomarkers were tested only for differentially abundant taxa (*p*_*adj*_ < 0.05) using Spearman’s *ρ* [24]. Differences in blood glucose and plasma insulin levels in mice were tested by two-way ANOVA, repeated measurements with Bonferroni post hoc test, and differences in fasting blood glucose were tested for by Wilcoxon rank ∑ tests.

## Results

Individuals with prediabetes displayed higher fasting plasma levels of glucose, insulin, C-peptide, triacylglycerol and hsCRP, HbA_1c_, HOMA-IR, BMI, and waist circumference compared with individuals with normal glucose regulation (Table 1). Individuals with prediabetes recruited from IMI-DIRECT and DanFunD were similar with respect to clinical biomarkers (ESM Table 1). Among 142 individuals from DanFunD where dietary habits could be reliably compared, intake of meat, poultry, fish and vegetables did not differ between individuals with prediabetes (*n* = 71) and normal glucose regulation (*n* = 71), whereas the intake of fruit was slightly higher among individuals with normal glucose regulation (ESM Table 2).

**Table 1:** Characteristics of the study population

	Normal glucose regulation	Prediabetes	*p* value
*n*	134	134	
Women, *n* (%)	53 (40)	53 (40)	1.0
Age, years	62 (55–67)	63 (57–68)	0.12
Fasting plasma glucose, mmol/l	5.2 (5.0–5.4)	6.3 (6.1–6.6)	<0.001
HbA_1c_, mmol/mol	34 (33–36)	38 (36–41)	<0.001
HbA_1c_, %	5.3 (5.2–5.4)	5.6 (5.5–5.9)	<0.001
Fasting plasma insulin, pmol/l^a^	50.0 (31.9–68.8)	78.3 (55.2–120.8)	<0.001
Plasma C-peptide, mmol/l	0.58 (0.45–0.71)	0.86 (0.69–1.08)	<0.001
HOMA-IR^a^	1.87 (1.20–2.66)	3.73 (2.45–5.60)	<0.001
Fasting plasma hsCRP, nmol/l^a^	7.43 (4.19–14.57)	13.81 (5.90–25.62)	<0.001
BMI, kg/m^2^	25.7 (23.5–27.5)	27.8 (25.0–30.9)	<0.001
Waist circumference, cm	90 (82–96)	100 (93–107)	<0.001
Fasting plasma triacylglycerol, mmol/l^a,b^	0.98 (0.83–1.29)	1.36 (0.97–1.93)	<0.001
Treatment for hypertension, *n* (%)	39 (29)	47 (35)	0.30
Treatment for hypercholesterolaemia, *n* (%)^b^	22 (16)	19 (14)	0.61

Data represent median (interquartile range) unless otherwise indicated

*p* values are from Wilcoxon rank ∑ tests for continuous variables and *χ*^2^ tests for categorical variables

^a^Plasma insulin, hsCRP, triacylglycerol and HOMA-IR were only available for 254, 267, 227 and 254 individuals, respectively

^b^Individuals who received treatment for hypercholesterolaemia were excluded from analyses of triacylglycerol levels

### Differences at genus and OTU levels

**Fig. 1:**
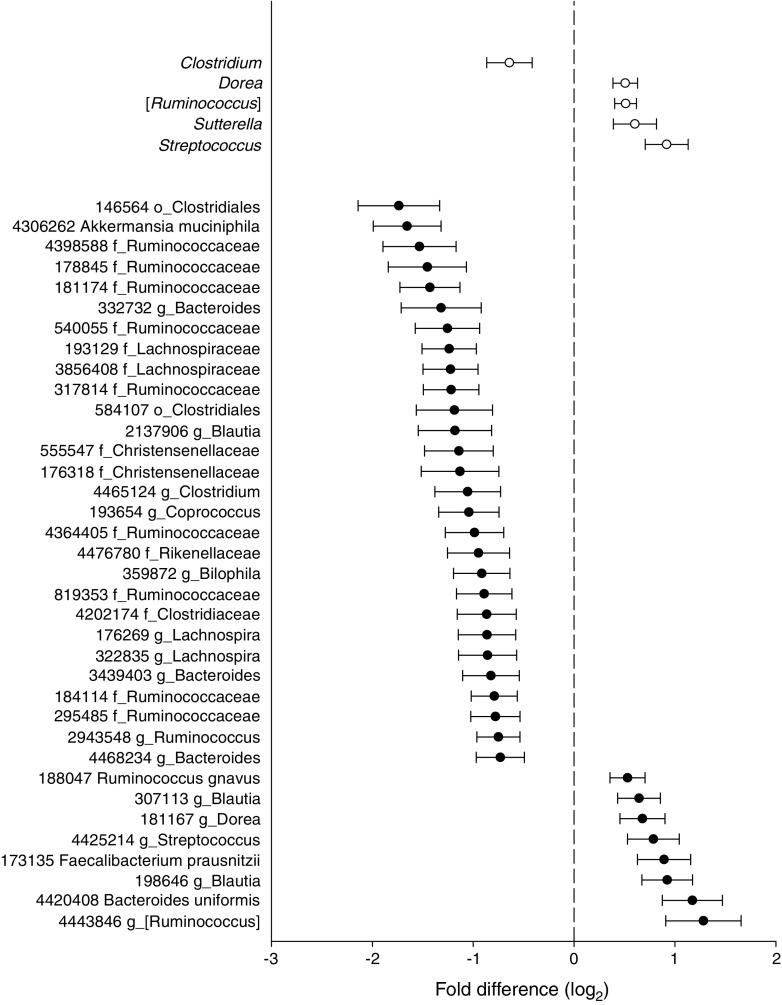
Genera and OTUs that display differential abundance among 134 individuals with prediabetes and 134 individuals with normal glucose regulation (*p*_*adj*_ < 0.05). Genera are depicted by white circles and OTUs by black circles. Circles indicate mean log_2_ fold difference and horizontal bars indicate SEM. Positive values imply higher abundance among individuals with prediabetes and negative values imply lower abundance among individuals with prediabetes. The taxa names indicate the lowest taxonomic affiliation available for the OTUs in the Greengenes database. To obtain a more specific affiliation we blasted the OTUs against the NCBI bacterial database, the best match with the per cent identity is provided in ESM Table 3

Five bacterial genera and 36 OTUs differed significantly in abundance between individuals with prediabetes and those with normal glucose regulation. At the genus level, the abundance of *Clostridium* was decreased, whereas the abundances of *Dorea*, [*Ruminococcus*], *Sutterella* and *Streptococcus* were increased among individuals with prediabetes (*p*_*adj*_ = 0.0497 to 1 × 10^−4^, Fig. 1 and ESM Table 3). At the OTU level, eight OTUs were increased and 28 were decreased among individuals with prediabetes (Fig. 1 and ESM Table 3). The two OTUs that differed the most between the two groups were classified as a member of the Clostridiales (OTU 146564) and *A. muciniphila*, which both displayed lower abundance among individuals with prediabetes (mean log_2_ fold difference −1.74 (SEM 0.41), *p*_*adj*_ = 2 × 10^−3^ and −1.65 (SEM 0.34), *p*_*adj*_ = 4 × 10^−4^, respectively). For *Faecalibacterium prausnitzii*, a prominent butyrate producer, we observed increased abundance of one strain but decreased abundance of another strain in individuals with prediabetes (ESM Table 3).

**Fig. 2:**
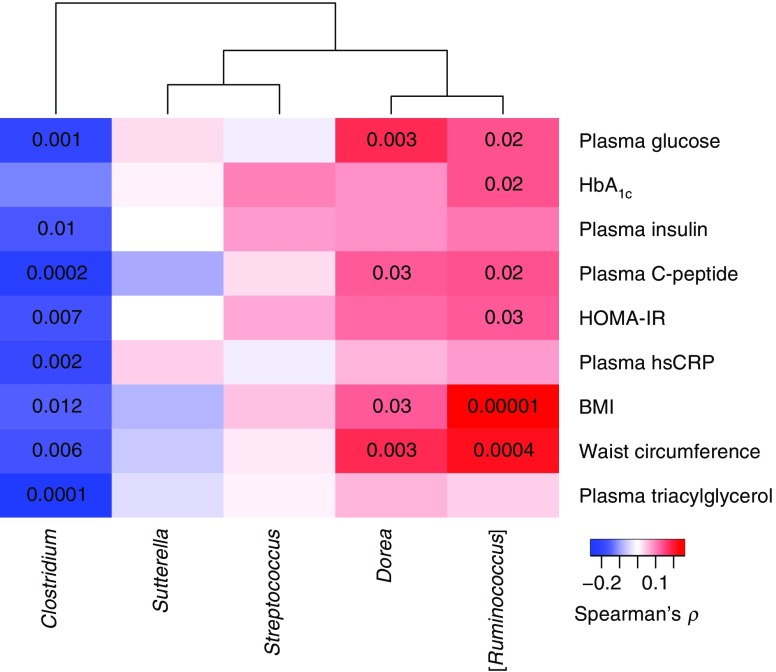
Association between differentially abundant genera and clinical biomarkers relevant for diabetes in the total group of 268 individuals. The genera names are from the Greengenes database. The colour key indicates Spearman’s *ρ* and the numbers in the cells represent *p* values <0.05. Spearman’s *ρ* and associated *p* values are listed in ESM Table 4

**Fig. 3:**
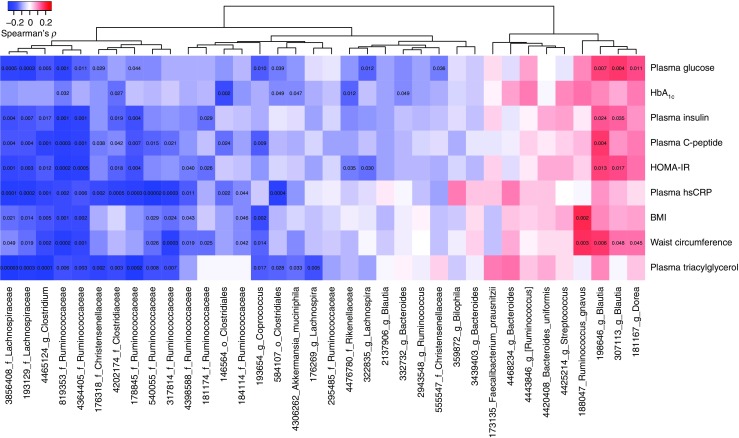
Association between differentially abundant OTUs and clinical biomarkers relevant for diabetes in the total group of 268 individuals. The taxa names indicate the lowest taxonomic affiliation available for the OTUs in the Greengenes database. To obtain a more specific affiliation we blasted the OTUs against the NCBI bacterial database, the best match with the per cent identity are provided in ESM Table 3. The colour key indicates Spearman’s *ρ* and the numbers in the cells represent *p* values <0.05. Spearman’s *ρ* and associated *p* values are listed in ESM Table 4

Next, we examined the correlations between the differentially abundant taxa and clinical biomarkers. The abundance of *Clostridium* was negatively correlated with fasting plasma levels of glucose, insulin, C-peptide, triacylglycerol and hsCRP, as well as HOMA-IR, BMI and waist circumference (*p* ≤ 0.01, Fig. 2 and ESM Table 4). Concordant with an increased abundance among individuals with prediabetes, [*Ruminococcus*] was positively correlated with fasting plasma levels of glucose and C-peptide, as well as HOMA-IR, HbA_1c_, BMI and waist circumference (*p* ≤ 0.03), whereas *Dorea* was positively correlated with fasting plasma glucose and C-peptide, and BMI and waist circumference (*p* ≤ 0.03). Notably, the OTUs displaying decreased abundance among individuals with prediabetes correlated most strongly with plasma triacylglycerol and hsCRP (Fig. 3 and ESM Table 4). Two OTUs (OTU 3856408 and OTU 193129) classified as *Lachnospiraceae* (96% identity to *Lachnobacterium bovis* strain LRC 5382), two OTUs (OTU 4364405 and OTU 819353) classified as *Ruminococcaceae* (92% identity to *Pseudoflavonifractor capillosus* strain ATCC 29799) and OTU 4465124 classified as *Clostridium* (ESM Table 3) correlated particularly strongly and negatively with fasting plasma levels of glucose, insulin, C-peptide, triacylglycerol and hsCRP, as well as HOMA-IR, BMI and waist circumference (Fig. 3 and ESM Table 4). *L. bovis* and *P. capillosus* belong to the *Clostridium* clusters XIVa and IV, respectively, which are known to contain multiple butyrate-producing bacteria. Two OTUs (OTU 198646 and OTU 307113) classified as *Blautia* sp. and mapping with 99% identity to *Blautia wexlerae* strain DSM 19850 (ESM Table 3) were positively correlated with fasting plasma levels of glucose and insulin, as well as HOMA-IR and waist circumference (Fig. 3 and ESM Table 4). OTU 188047, which mapped with 99% identity to *Dorea longicatena* strain 111–35, correlated positively with BMI and waist circumference. OTU 181167 classified as *Dorea* sp. and mapping with 99% identity to *Coprococcus comes* strain ATCC 27758 (ESM Table 3) was positively correlated with plasma glucose and waist circumference (Fig. 3).

### Microbiota composition and diversity

Overall, the bacterial community composition assessed by principal coordinates analysis (PCoA) based on unweighted UniFrac distances showed a weak association with prediabetes (*p* = 0.02, *r*^2^ = 0.006) (ESM Fig. 1a). PCoA based on weighted UniFrac (*p* = 0.35, *r*^2^ = 0.004) and Bray–Curtis distances (*p* = 0.19, *r*^2^ = 0.004) did not show any clustering (ESM Fig. 1b,c). As unweighted UniFrac, in contrast to weighted UniFrac, does not take abundance into account, these results demonstrate that the weak association between prediabetes and gut microbiota is driven by the presence/absence status of low abundant taxa.

**Fig. 4:**
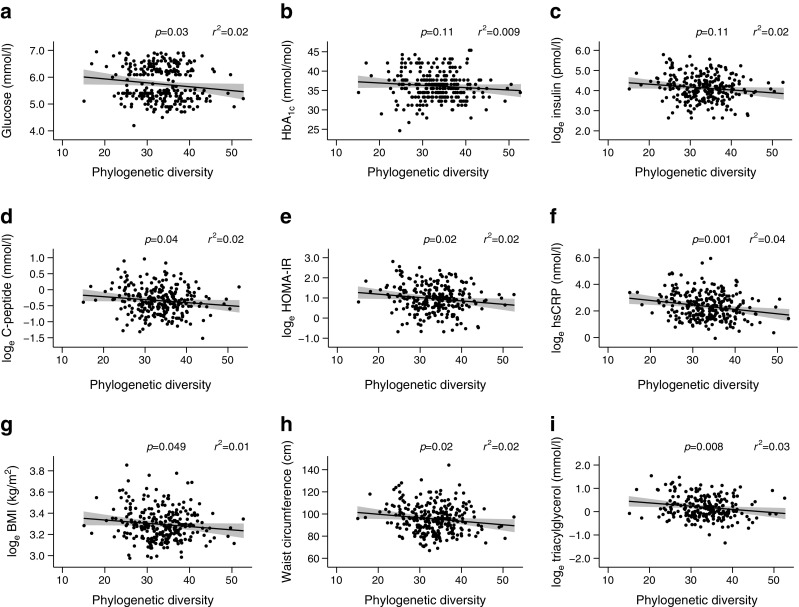
Association between clinical biomarkers and α-diversity estimated as phylogenetic diversity in the total group of 268 individuals. The black line represents the fitted regression line and the grey shaded areas represent the 95% CIs. *r*^2^ is the proportion of variance in the clinical biomarkers that can be predicted from the phylogenetic diversity

Bacterial richness estimated as the number of observed OTUs was 543 ± 92 and 562 ± 93 (mean ± SEM) among individuals with prediabetes and normal glucose regulation, respectively (*p* = 0.09, ESM Fig. 2a). Corresponding values for α-diversity estimated as phylogenetic diversity were 33 ± 0.49 and 34 ± 0.51 (mean ± SEM), respectively (*p* = 0.06, ESM Fig. 2b). In the total group of 268 individuals, α-diversity was negatively associated with clinical biomarkers related to type 2 diabetes: the higher the α-diversity, the lower the fasting plasma levels of glucose, insulin, C-peptide, triacylglycerol and hsCRP, as well as HOMA-IR, BMI and waist circumference (*p* = 0.049–0.001; Fig. 4 and ESM Table 5). Plasma triacylglycerol and hsCRP were the clinical biomarkers which were most strongly associated with α-diversity.

### Transfer of gut microbiota to conventional and germfree mice

To assess whether the aberrant microbiota could directly modulate host glucose metabolism, we transferred faecal microbiota from human donors to conventional C57BL/6 J mice. Prior to transfer, we pooled faeces from four individuals with fasting plasma glucose >6.1 mmol/l and HbA_1c_ >42 mmol/mol (6.0%) and from four sex-matched individuals with normal levels of fasting plasma glucose. Mean ± SD fasting plasma glucose and HbA_1c_ were 6.4 ± 0.16 mmol/l and 44 ± 0.5 mmol/mol (6.2 ± 0.05%), respectively, among donors with prediabetes, and 4.5 ± 0.12 mmol/l and 33 ± 0.9 mmol/mol (5.2 ± 0.08%), respectively, among donors with normal glucose regulation. We observed that the prediabetic phenotype did not precipitate in recipient mice possibly due to unsuccessful colonisation of key microbial taxa in the conventional mice as a result of antagonism from the indigenous mouse microbiota (ESM Fig. 3).

In an attempt to avoid competition by the indigenous mouse gut microbiota, we colonised germfree Swiss Webster mice and used a ‘personalised’ approach consisting of the transfer of stool samples from selected individual donors rather than pooled samples. First, we transferred the faecal microbiota of a male donor with screen-detected, treatment-naive type 2 diabetes (two measurements of fasting plasma glucose >7.0 mmol/l) (case donor 1) and a BMI-matched male donor with normal glucose regulation (control donor 1). Faecal transfer did not result in impaired glucose regulation (ESM Fig. 4). Interestingly, mice transplanted with faeces from the donor with screen-detected type 2 diabetes had lower fasting blood glucose (ESM Fig. 4c, *p* = 0.049), possibly due to the lack of colonisation of key taxa, such as potentially protective strains belonging to the Clostridiales in the control recipients (ESM Table 6 and ESM Fig. 4l). We also performed a second transplantation experiment with faeces from another male donor with screen-detected, treatment-naive type 2 diabetes (120 min plasma glucose >11.1 mmol/l) (case donor 2) and a BMI-discrepant male donor with normal glucose regulation (control donor 2). This experiment showed no difference in blood glucose levels in the fasting state or during GTT between the two groups of mice (ESM Fig. 4d–e). Furthermore, consistent with these results, the microbiota analysis showed low similarity of overall microbiota composition between recipient mice and the control donor, as well as a lack of colonisation of key microbial taxa (ESM Fig. 4 and ESM Table 6).

## Discussion

We have shown that individuals with prediabetes, a precursor state of type 2 diabetes and ischaemic cardiovascular disease, have aberrant intestinal microbiota characterised by a decreased abundance of the genus *Clostridium* and the mucin-degrading bacterium *A. muciniphila*.

Previous findings on the abundance of *A. muciniphila* among people with prediabetes or overt type 2 diabetes have been contradictory. Some studies have reported no difference [3], others increased abundance [5] and others decreased abundance of *A. muciniphila* [7]. It is most likely that these discrepancies are related to the observation in mice that metformin increases the levels of *Akkermansia* [25]; a finding which was recently supported by two studies showing higher abundance of *A. muciniphila* in participants taking metformin [26, 27]. Interestingly, it has been shown that oral administration of *A. muciniphila* improves glucose tolerance and insulin resistance, and reduces adipose tissue inflammation in mice [25, 28], possibly via Toll-like receptor 2 signalling [29].

At the genus level, *Clostridium* was depleted among individuals with prediabetes and negatively correlated with fasting levels of glucose and triacylglycerol as well as estimates of insulin resistance, inflammation and adiposity. These findings correspond well with a previous study reporting decreased abundance of *Clostridium* species among individuals with type 2 diabetes and a negative correlation between *Clostridium* species and plasma levels of glucose, insulin, C-peptide and triacylglycerol [3]. Concordant with previous studies consistently reporting depletion of butyrate-producing bacteria in individuals with type 2 diabetes [3, 5, 9], we found that individuals with prediabetes have decreased abundance of several bacteria with the potential to produce butyrate. However, for one of the most prominent butyrate producers, *F. prausnitzii*, our results were ambiguous with increased abundance of one strain but decreased abundance of another strain. Moreover, it remains unknown whether the increase in a subset of bacteria with the potential for butyrate production translates to an actual increased production of butyrate in humans in vivo. Among the few OTUs that were increased in individuals with prediabetes and positively correlated with biomarkers of poor metabolic health, we identified *D. longicatena* and *C. comes*, both of which have been demonstrated recently to be associated with obesity and several markers of dysmetabolism [30]. Interestingly, *D. longicatena* has genes for the degradation of human mucins, and *D. longicatena* and *C. comes* both produce glutamate, which may be related to the prevalence of obesity [30].

Our finding that increasing species diversity was associated with improved glucose regulation and less adiposity and inflammation is in accordance with previous findings, where low bacterial richness has been associated with adiposity, insulin resistance, dyslipidaemia and inflammation [31]. Interestingly, we demonstrated the strongest correlations for plasma triacylglycerol and hsCRP. Notably, estimates of decreased bacterial richness have been observed among metformin-naive individuals with type 2 diabetes but not among those treated with metformin [9]. In contrast to the present and previous findings, a Finnish study reported that richness was positively associated with HbA_1c_ [32], and a study of mice transplanted with faeces from obese and lean individuals showed a positive correlation of OTU richness with both HOMA-IR and fasting insulin [33]. In the present study we did not include stimulated measurements of plasma glucose and insulin, precluding assessment of gut microbiota differences between states of impaired fasting glucose and impaired glucose tolerance [34].

Some of the microbes that we found to differ between individuals with prediabetes and normal glucose regulation have previously been related to other disorders including inflammatory bowel disease [35], atopic dermatitis [36] and colorectal adenoma [37]. Therefore, although major differences exist in host genetics and disease phenotype expression, we conclude that some of the microbial compositional changes that we demonstrate in individuals with prediabetes are common to several seemingly diverse pathological phenotypes characterised by low-grade inflammation. Recently, we proposed a ‘common ground hypothesis’ on the potential role of aberrant intestinal microbiota in the pathogenesis of chronic polygenic disorders. Briefly, this hypothesis suggests that an aberrant gut microbiota may trigger genetic susceptibility to a spectrum of chronic disorders and thus directly contribute to elicit specific diseases in predisposed individuals [38].

In the present study we also examined whether the aberrant gut microbiota were causally involved in prediabetes by transplanting human faecal microbiota into mice. We were, however, unable to transfer the prediabetic phenotype to mice. The lack of phenotypic transmission may have multiple explanations. One explanation could be that the gut microbiota alterations in prediabetes were too subtle compared with substantial rearrangements, such as those induced by bariatric surgery and metformin, that have been causally linked to metabolic phenotypes [27, 39]. Another explanation could be that the colonisation efficacy of key species may be too weak, as we show in our study and as recently reported in comparable experiments [33, 40]. Moreover, the lack of cross-species transferability may be attributed to considerable differences in the composition of nutrients of mouse and human diets, host-specificity of the immune response [41] or competition with the indigenous microbiota in case of the transfers to conventional mice.

In conclusion, individuals with prediabetes, who as a group have dysglycaemia, low-grade inflammation, insulin resistance, hypertriacylglycerolaemia and overweight, present aberrant intestinal microbiota with the most significant signature being depletion of the genus *Clostridium* and the mucin-degrading bacterium *A. muciniphila*. Our findings are comparable with observations in overt chronic diseases characterised by low-grade inflammation, such as inflammatory bowel disease, colorectal adenoma and treatment-naive type 2 diabetes, suggesting that shared gut microbial alterations may be a signature of low-grade inflammation.

## Electronic supplementary material

ESM (PDF 643 kb)

ESM Tables 3 & 4 (XLSX 35 kb)

## Data Availability

The datasets generated and analysed during the current study are not publicly available as they contain information that could compromise research participant privacy, but they are available from the corresponding authors on reasonable request.

## Abbreviations

DanFunD: Danish study of Functional Disorders
hsCRP: High-sensitivity C-reactive protein
IMI-DIRECT: Innovative Medicines Initiative – DIabetes REseraCh on patient straTification
OTU: Operational taxonomic unit
PCoA: Principal coordinates analysis
