# Supplementary material for: Aberrant intestinal microbiota in individuals with prediabetes
Source: Diabetologia. 2018 Jan 29;61(4):810–20. doi: 10.1007/s00125-018-4550-1 (PMC6448993; doi:10.1007/s00125-018-4550-1)
Supplement: Supplementary file 1 — (PDF 643 kb) [file 125_2018_4550_MOESM1_ESM.pdf]

Electronic Supplementary Material

ESM Methods Supplementary information about amplification of 16S rRNA genes and analysis of quality filtered reads

ESM Table 1 Characteristics of the study population stratified by sub-cohort

ESM Table 2 Diet in sub-cohort 1

ESM Table 3 Genera and operational taxonomic units that display differential abundance among individuals with prediabetes and normal glucose regulation

ESM Table 4 Association between differentially abundant genera and operational taxonomic units and clinical biomarkers relevant for diabetes

ESM Table 5 Associations between α-diversity estimated as phylogenetic diversity and clinical biomarkers

ESM Table 6. List of the 23 OTUs that were found as highly abundant in the inocula and germfree Swiss Webster recipient mice

ESM Fig. 1 Principal coordinate analysis of the overall composition of the faecal microbiota

ESM Fig. 2 Species richness in the faecal microbiota

ESM Fig. 3 Faecal transfer from human donors with distinct glucose regulation in conventional C57Bl/6J mice

ESM Fig. 4 Faecal transfer from human donors with distinct glucose regulation in germfree Swiss Webster mice

ESM Methods

Supplementary information about amplification of 16S rRNA genes

16S rRNA genes were amplified in duplicate reactions in volumes of 25 µl containing 1x Five Prime Hot Master Mix (5 PRIME GmbH), 200 nM of each primer, 0.4 mg/ml BSA, 5% DMSO and 20 ng of total faecal genomic DNA. PCR was carried out under the following conditions: initial denaturation for 3 min at 94°C, followed by 25 cycles of denaturation for 45 sec at 94°C, annealing for 60 sec at 52°C and elongation for 90 sec at 72°C, and a final elongation step for 10 min at 72°C. Duplicates were combined, purified with the NucleoSpin Gel and PCR Clean-up kit (Macherey-Nagel) and quantified using the Quant-iT PicoGreen dsDNA kit (Invitrogen). Purified PCR products were diluted to 5 ng/µl and pooled in equal amounts. The pooled amplicons were purified again using Ampure magnetic purification beads (Agencourt) to remove short amplification products.

Supplementary information about analysis of quality filtered reads

Sequences were clustered into operational taxonomic units (OTUs) at a 97% identity threshold using an open-reference OTU picking approach with UCLUST [1] against the Greengenes reference database [2] (13_8 release). All sequences that failed to cluster when tested against the Greengenes database were used as input for picking OTUs de novo. Representative sequences for the OTUs were Greengenes reference sequences or cluster seeds, and were taxonomically assigned using the Greengenes taxonomy and the Ribosomal Database Project Classifier [3]. Representative OTUs were aligned using PyNAST [4] and used to build a phylogenetic tree with FastTree [5], which was used to calculate α- and β-diversity of samples using Phylogenetic Diversity [6] and UniFrac [7]. Three-dimensional principal coordinates analysis (PCoA) plots were visualized using Emperor [8]. Chimeric sequences were identified with ChimeraSlayer [9].

References

[1] Edgar RC (2010) Search and clustering orders of magnitude faster than BLAST. Bioinformatics 26: 2460-2461

[2] DeSantis TZ, Hugenholtz P, Larsen N, et al. (2006) Greengenes, a Chimera-Checked 16S rRNA Gene Database and Workbench Compatible with ARB. Applied and Environmental Microbiology 72: 5069-5072

[3] Wang Q, Garrity GM, Tiedje JM, Cole JR (2007) Naïve Bayesian Classifier for Rapid Assignment of rRNA Sequences into the New Bacterial Taxonomy. Applied and Environmental Microbiology 73: 5261-5267

[4] Caporaso JG, Bittinger K, Bushman FD, DeSantis TZ, Andersen GL, Knight R (2010) PyNAST: a flexible tool for aligning sequences to a template alignment. Bioinformatics 26: 266-267

[5] Price MN, Dehal PS, Arkin AP (2010) FastTree 2--approximately maximum-likelihood trees for large alignments. PLoS One 5: e9490

[6] Faith DP (1992) Conservation evaluation and phylogenetic diversity. Biological Conservation 61: 1-10

[7] Lozupone C, Knight R (2005) UniFrac: a New Phylogenetic Method for Comparing Microbial Communities. Applied and Environmental Microbiology 71: 8228-8235

[8] Vázquez-Baeza Y, Pirrung M, Gonzalez A, Knight R (2013) EMPeror: a tool for visualizing high-throughput microbial community data. GigaScience 2: 1-4

[9] Haas BJ, Gevers D, Earl AM, et al. (2011) Chimeric 16S rRNA sequence formation and detection in Sanger and 454-pyrosequenced PCR amplicons. Genome Research 21: 494-504

ESM Table 1 Characteristics of the study population stratified by sub-cohort

	Sub-cohort 1		Sub-cohort 2	
	Normal glucose regulation	Prediabetes	Normal glucose regulation	Prediabetes
No.	71	71	63	63
Women, no. (%)	21 (30)	21 (30)	32 (51)	32 (51)
Age, years	61 (55;66)	63 (57;67)	62 (56;68)	64 (57;69)
Fasting plasma glucose, mmol/l	5.2 (4.9;5.4)	6.3 (6.1;6.5)	5.3 (5.0;5.4)	6.4 (6.2;6.6)
HbA_{1c}, mmol/mol	34 (33;36)	37 (34;39)	34 (33;36)	39 (37;42)
HbA_{1c}, %	5.3 (5.2;5.5)	5.5 (5.3;5.7)	5.3 (5.2;5.4)	5.8 (5.6;6.0)
Fasting plasma insulin, pmol/l	50.0 (30.6;69.5)	84.7 (55.6;128)	49.7 (33.7;65.6)	72.6 (55.2;107)
Fasting plasma C-peptide, mmol/l	0.60 (0.42;0.73)	0.80 (0.65;1.08)	0.56 (0.45;0.71)	0.92 (0.75;1.14)
HOMA-IR	2.00 (1.18;2.75)	4.05 (2.56;6.21)	1.86 (1.30;2.60)	3.66 (2.45;5.14)
Fasting plasma hsCRP, nmol/l	6.67 (4.10;14.57)	13.43 (5.05;28.38)	7.71 (4.48;14.86)	15.52 (6.38;24.67)
BMI, kg/m^2	26.2 (24.1;27.7)	27.0 (24.5;30.1)	25.0 (23.0;27.2)	28.7 (26.0;31.1)
Waist circumference, cm	91 (86;98)	96 (89;104)	86 (80;96)	102 (98;109)
Fasting plasma triacylglycerol, mmol/l	0.95 (0.82;1.30)	1.29 (0.90;1.92)	1.02 (0.84;1.28)	1.36 (1.07;1.93)
Treatment for hypertension, no (%)	22 (31)	28 (39)	17 (27)	19 (30)
Treatment for hypercholesterolemia, no. (%)	10 (14)	11 (15)	12 (19)	8 (13)

Sub-cohort 1: individuals with prediabetes were recruited from the DanFunD study.
Sub-cohort 2: individuals with prediabetes were recruited from the Danish part of the IMI-DIRECT study.
Individuals with normal glucose regulation were recruited only from the DanFunD and matched to individuals with prediabetes with respect to sex and age (n=134).
Values are median (IQR) unless otherwise indicated. Individuals who received treatment for hypercholesterolemia were excluded from analyses of triacylglycerol levels. Plasma insulin, HOMA-IR, hsCRP, and triacylglycerol were only available for 254, 254, 267, and 227 individuals, respectively.

ESM Table 2 Diet in sub-cohort 1

	Normal glucose regulation (n=71)	Prediabetes (n = 71)	*p* value
Meat (beef, veal, pork, or lamb), servings per week	3.5 (1.5-3.5)	3.5 (1.5-6.0)	0.07
Poultry, servings per week	1.5 (1.5-1.5)	1.5 (1.5-1.5)	0.23
Fish, servings per week	1.5 (1.5-1.5)	1.5 (0.0-1.5)	0.19
Vegetables, servings per day	1.7 (0.9-2.1)	1.5 (1.1-2.1)	0.77
Fruit, servings per day	1.5 (1.5-3.5)	1.5 (0.6-1.5)	0.02

All individuals were recruited from the DanFunD study.
Numbers are median (IQR). *p* values are from Wilcoxon rank sum tests.

ESM Table 3 and ESM Table 4. See Excel sheet: ESM Table 3 and 4

ESM Table 5 Associations between α-diversity estimated as phylogenetic diversity and clinical biomarkers

	n	Effect estimate (95% CI)	*p* value
Fasting plasma glucose, mmol/l	268	-0.01 (-0.03 to -0.001)	0.03
HbA_{1c}, mmol/mol	268	-0.06 (-0.14 to 0.01)	0.11
HbA_{1c}, %	268	-0.06 (-0.14 to 0.01)	0.11
Log(plasma insulin, pmol/l)	254	-0.01 (-0.03 to -0.001)	0.03
Log(plasma C-peptide, mmol/l)	268	-0.009 (-0.02 to -0.0006)	0.04
Log(HOMA-IR)	254	-0.02 (-0.03 to -0.002)	0.02
Log(plasma C-reactive protein, nmol/l)	267	-0.03 (-0.05 to -0.01)	0.001
Log(BMI, kg/m^2)	268	-0.003 (-0.006 to -0.00001)	0.049
Waist circumference, cm	268	-0.32 (-0.58 to -0.06)	0.02
Log(plasma triacylglycerol, mmol/l)	227	-0.01 (-0.02 to -0.004)	0.008

Effect estimates and *p* values are from linear regression analyses. Effect estimates represent the effect of a one unit increase in α-diversity on the clinical biomarkers.

ESM Table 6. List of the 23 OTUs that were found as highly abundant in the inocula and germfree Swiss Webster recipient mice

Taxonomy	OTU	Increased in (human cohort)	Increased in experiment 1 (GF recipients)	Increased in experiment 2 (GF recipients)
k_Bacteria; p_Bacteroidetes; c_Bacteroidia; o_Bacteroidales; f_Bacteroidaceae; g_Bacteroides; s_	3439403	Controls	ns	* (Cases)
k_Bacteria; p_Bacteroidetes; c_Bacteroidia; o_Bacteroidales; f_Bacteroidaceae; g_Bacteroides; s_	332732	Controls	* (Cases)	** (Cases)
k_Bacteria; p_Bacteroidetes; c_Bacteroidia; o_Bacteroidales; f_Bacteroidaceae; g_Bacteroides; s_	4468234	Controls	ns	ns
k_Bacteria; p_Bacteroidetes; c_Bacteroidia; o_Bacteroidales; f_Rikenellaceae; g_; s_	4476780	Controls	ns	** (Controls)
k_Bacteria; p_Firmicutes; c_Clostridia; o_Clostridiales; f_; g_; s_	146564	Controls	ns	ns
k_Bacteria; p_Firmicutes; c_Clostridia; o_Clostridiales; f_; g_; s_	584107	Controls	ns	ns
k_Bacteria; p_Firmicutes; c_Clostridia; o_Clostridiales; f_Clostridiaceae; g_; s_	4202174	Controls	ns	ns
k_Bacteria; p_Firmicutes; c_Clostridia; o_Clostridiales; f_Clostridiaceae; g_Clostridium; s_	4465124	Controls	ns	ns
k_Bacteria; p_Firmicutes; c_Clostridia; o_Clostridiales; f_Lachnospiraceae; g_Blautia; s_	2137906	Controls	ns	0.06 (Controls)
k_Bacteria; p_Firmicutes; c_Clostridia; o_Clostridiales; f_Lachnospiraceae; g_Lachnospira; s_	176269	Controls	ns	ns
k_Bacteria; p_Firmicutes; c_Clostridia; o_Clostridiales; f_Ruminococcaceae; g_; s_	819353	Controls	ns	* (Controls)

k_Bacteria; p_Firmicutes; c_Clostridia; o_Clostridiales; f_Ruminococcaceae; g_; s_	4364405	Controls	ns	ns
k_Bacteria; p_Firmicutes; c_Clostridia; o_Clostridiales; f_Ruminococcaceae; g_; s_	184114	Controls	ns	* (Controls)
k_Bacteria; p_Firmicutes; c_Clostridia; o_Clostridiales; f_Ruminococcaceae; g_; s_	181174	Controls	ns	ns
k_Bacteria; p_Firmicutes; c_Clostridia; o_Clostridiales; f_Ruminococcaceae; g_; s_	540055	Controls	ns	ns
k_Bacteria; p_Firmicutes; c_Clostridia; o_Clostridiales; f_Ruminococcaceae; g_; s_	178845	Controls	ns	ns
k_Bacteria; p_Firmicutes; c_Clostridia; o_Clostridiales; f_Ruminococcaceae; g_Ruminococcus; s_	2943548	Controls	ns	ns
k_Bacteria; p_Proteobacteria; c_Deltaproteobacteria; o_Desulfovibrionales; f_Desulfovibrionaceae; g_Bilophila; s_	359872	Controls	* (Controls)	* (Cases)
k_Bacteria; p_Verrucomicrobia; c_Verrucomicrobiae; o_Verrucomicrobiales; f_Verrucomicrobiaceae; g_Akkermansia; s_muciniphila	4306262	Controls	ns	** (Cases)
k_Bacteria; p_Firmicutes; c_Bacilli; o_Lactobacillales; f_Streptococcaceae; g_Streptococcus; s_	4425214	Cases	ns	ns
k_Bacteria; p_Firmicutes; c_Clostridia; o_Clostridiales; f_Lachnospiraceae	4443846	Cases	ns	ns
k_Bacteria; p_Firmicutes; c_Clostridia; o_Clostridiales; f_Lachnospiraceae; g_[Ruminococcus]; s_gnavus	188047	Cases	ns	* (Controls)
k_Bacteria; p_Firmicutes; c_Clostridia; o_Clostridiales; f_Lachnospiraceae; g_Dorea; s_	181167	Cases	ns	* (Controls)

* p<0.05, ** p<0.01
GF: Germfree; ns: non-significant

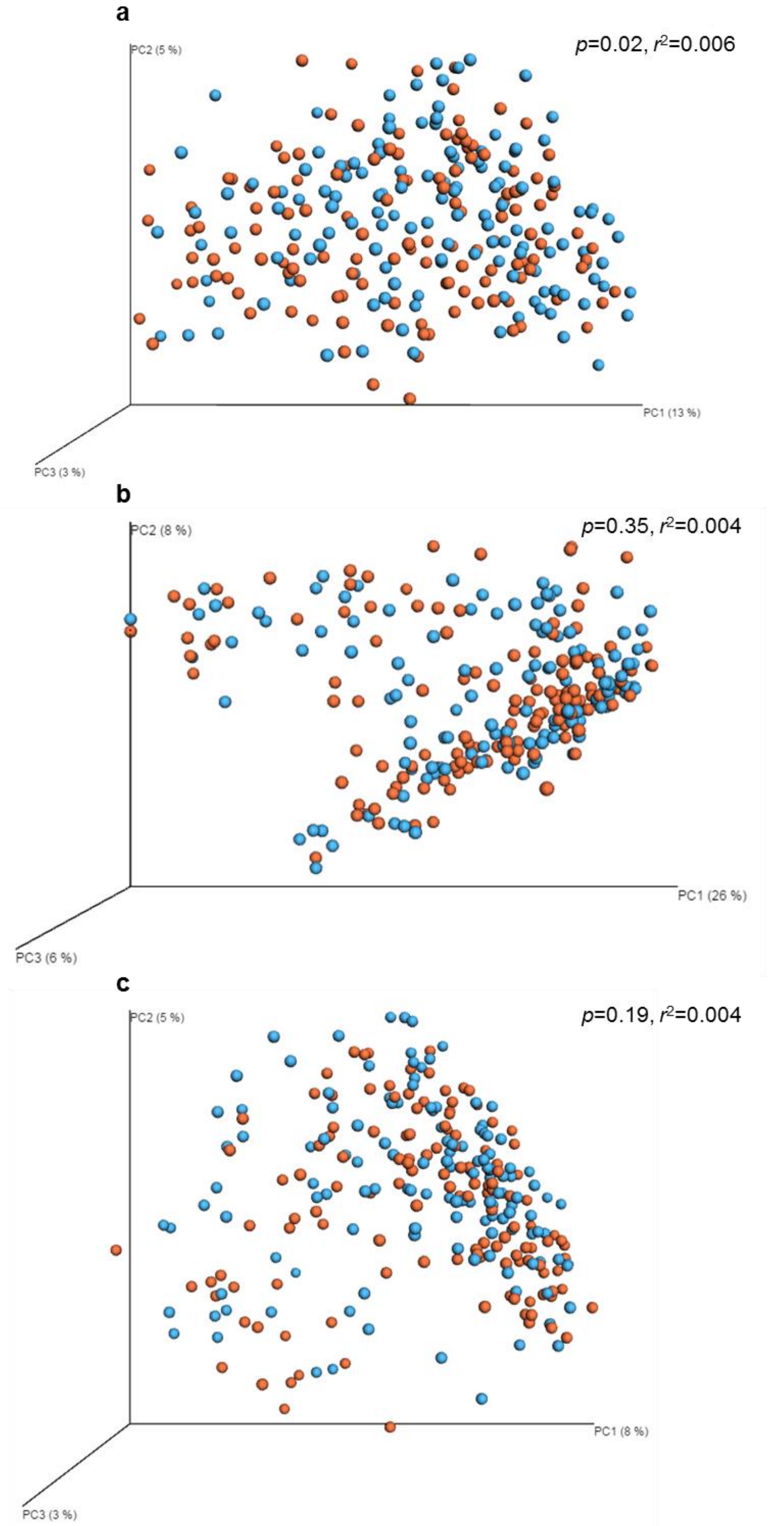

ESM Fig. 1 Principal coordinate analysis of the overall composition of the faecal microbiota
The principal coordinate analysis is based on **a**) unweighted UniFrac, **b**) weighted UniFrac and **c**) Bray-Curtis distances. Blue dots represent normal glucose regulation and red dots represent impaired glucose regulation. p values are from multivariate non-parametric analysis of variance implemented in the Adonis function (999 permutations) in the R vegan package.

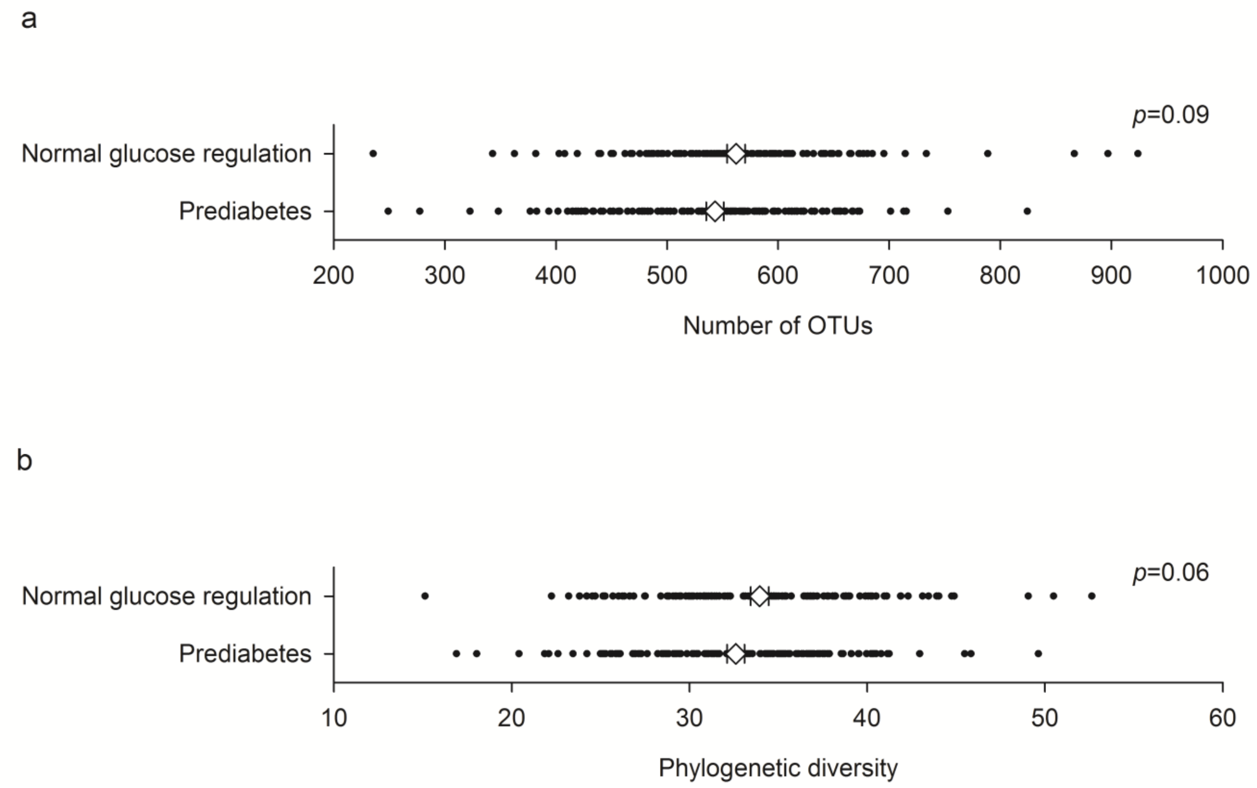

ESM Fig. 2 Species richness in the faecal microbiota
Species richness was estimated as number of OTUs (**a**) and phylogenetic diversity (**b**). Diamonds represent the mean and horizontal bars represent ± SEM.

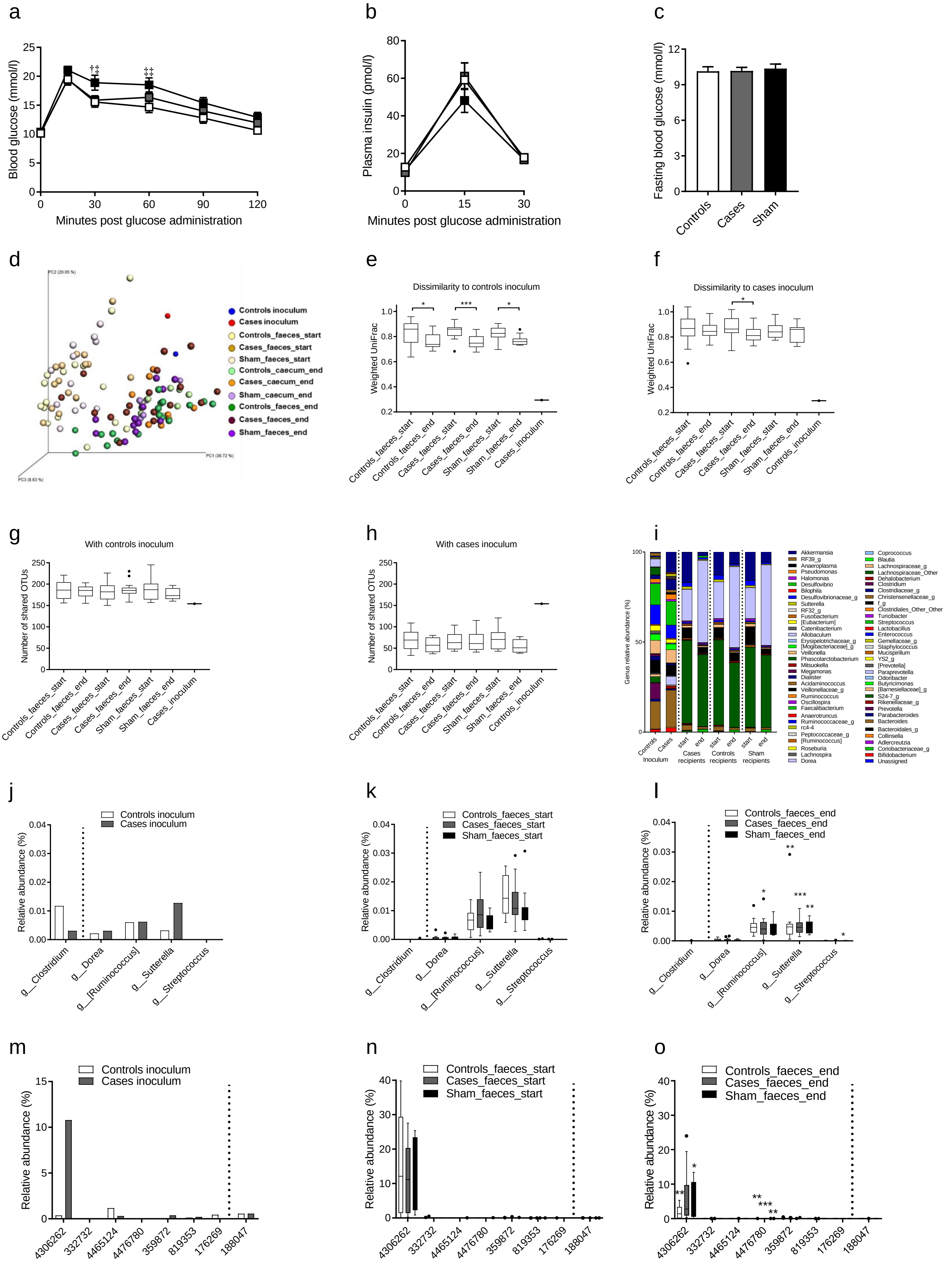
a
Blood glucose (mmol/l)
Minutes post glucose administration
b
Plasma insulin (pmol/l)
Minutes post glucose administration
c
Fasting blood glucose (mmol/l)
Controls
Cases
Sham
d
Controls inoculum
Cases inoculum
Controls_faeces_start
Cases_faeces_start
Sham_faeces_start
Controls_caecum_end
Cases_caecum_end
Sham_caecum_end
Controls_faeces_end
Cases_faeces_end
Sham_faeces_end
e
Dissimilarity to controls inoculum
Weighted UniFrac
Controls_faeces_start
Controls_faeces_end
Cases_faeces_start
Cases_faeces_end
Sham_faeces_start
Sham_faeces_end
Cases_inoculum
f
Dissimilarity to cases inoculum
Weighted UniFrac
Controls_faeces_start
Controls_faeces_end
Cases_faeces_start
Cases_faeces_end
Sham_faeces_start
Sham_faeces_end
Controls_inoculum
g
With controls inoculum
Number of shared OTUs
Controls_faeces_start
Controls_faeces_end
Cases_faeces_start
Cases_faeces_end
Sham_faeces_start
Sham_faeces_end
Cases_inoculum
h
With cases inoculum
Number of shared OTUs
Controls_faeces_start
Controls_faeces_end
Cases_faeces_start
Cases_faeces_end
Sham_faeces_start
Sham_faeces_end
Controls_inoculum
i
Genus relative abundance (%)
Controls
Cases
Inoculum
start
end
Cases recipients
Controls recipients
Sham recipients
j
Controls inoculum
Cases inoculum
Relative abundance (%)
g__Clostridium
g__Dorea
g__[Ruminococcus]
g__Sutterella
g__Streptococcus
k
Controls_faeces_start
Cases_faeces_start
Sham_faeces_start
Relative abundance (%)
g__Clostridium
g__Dorea
g__[Ruminococcus]
g__Sutterella
g__Streptococcus
l
Controls_faeces_end
Cases_faeces_end
Sham_faeces_end
Relative abundance (%)
g__Clostridium
g__Dorea
g__[Ruminococcus]
g__Sutterella
g__Streptococcus
m
Controls inoculum
Cases inoculum
Relative abundance (%)
4306262
332732
4465124
4476780
359872
819353
176269
188047
n
Controls_faeces_start
Cases_faeces_start
Sham_faeces_start
Relative abundance (%)
4306262
332732
4465124
4476780
359872
819353
176269
188047
o
Controls_faeces_end
Cases_faeces_end
Sham_faeces_end
Relative abundance (%)
4306262
332732
4465124
4476780
359872
819353
176269
188047

ESM Fig. 3 Faecal transfer from human donors with distinct glucose regulation in conventional C57Bl/6J mice.

(**a**) Glucose tolerance test of conventional C57Bl/6J mice fed Western diet and gavaged with faecal microbiota from four pooled donors with prediabetes (cases, n = 17), four pooled individuals with normal glucose regulation (controls, n = 17), or PBS (sham, n = 12) for four weeks; (**b**); Glucose stimulated insulin secretion during glucose tolerance test on mice from a; (**c**) Fasting blood glucose derived immediately before the glucose tolerance test described in a. Data are presented as mean ± SE and statistical evaluation was performed by two-way ANOVA, repeated measurements with Bonferroni post hoc test (a-b) and Wilcoxon rank-sum tests (c). † = $p < 0.05$ between cases and sham, ‡ and ‡‡ = $p < 0.05$ and < 0.01, respectively, between controls and sham. White colour: controls. Grey colour: cases. Black colour: sham.
(**d**) Principal coordinate analysis (PCoA) plot of faecal microbiota composition in the C57Bl/6J transplanted mice based on weighted UniFrac. The dots in the PCoA plot indicate either faecal samples collected from each mouse before (faeces_start) and at the end of the four-week gavage period (faeces_end) or caecal samples collected at the termination of the experiment (caecum_end); the values in brackets on the axes show the percentage of variation explained by the first three principal coordinates (PC). Gavage of human microbiota and sham treatment significantly shifted the composition of the microbiota ($p = 0.001$, $r^2 = 0.314$; Adonis 999 permutations). However, we observed no clustering of samples according to the type of inoculum ($p = 0.628$, $r^2 = 0.014$; Adonis 999 permutations), indicating that sham treatment and gavage of human microbiota had similar effects on the gut microbiota in the recipient mice. (**e-f**) Tukey box plots showing weighted UniFrac distances calculated to measure the dissimilarity of overall microbiota composition between (**e**) mouse faecal samples and controls inoculum and (**f**) mouse faecal samples and cases inoculum (0=identical; 1=completely different). At the end of the experiment, all faecal samples including those of sham mice became more similar to the controls inoculum, while only the faecal samples of mice that received human microbiota from cases became more similar to the cases inoculum (* $p< 0.05$ and *** $p< 0.001$ according to Wilcoxon matched-pairs signed rank test). (**g-h**) Tukey box plots showing the number of OTUs shared between (**g**) mouse faecal samples and controls inoculum and (**h**) mouse faecal samples and cases inoculum. The results show that gavage treatment *per se* significantly shifted the overall composition of the gut microbiota, and that more OTUs were shared between mouse faecal samples and controls inoculum. These results might indicate a more successful transfer of OTUs from the controls inoculum, which could also depend on its higher richness (OTUs in the controls inoculum = 346; OTUs in the cases inoculum = 202; OTUs shared between controls inoculum and cases inoculum = 154). (**i**) Bar plots showing the mean relative abundance of all microbial genera (n=68) identified in the faecal and caecal samples of the transplanted mice before (start) and at the end of the four-week gavage period (end). (**j**) Bar plots showing the relative abundance in the cases and control inocula of the five microbial genera that differed significantly in the human cohort. (**k-l**) Tukey box plots indicating the relative abundance of the five genera in the mouse faecal microbiota (**k**) before (faeces_start) and (**l**) at the end of the four-week gavage period (faeces_end). Stars (*) indicate significantly different abundance for OTUs at the end of the gavage period compared to before (* $p <0.05$, ** $p< 0.01$ and *** $p <0.001$ according to Wilcoxon matched-pairs signed rank test). (**m**) Bar plots showing the relative abundance in the cases and control inocula of the microbial OTUs that differed significantly in the human cohort; only 8 out of the 36 OTUs that differed significantly in the human cohort were found among the highly abundant OTUs present in the inocula and in the mouse samples (7 OTUs, on the left of the dotted line, had increased abundance in the controls in the human cohort while 1 OTU, on the right of the dotted line, had increased abundance in the cases in the human cohort (ESM Table 3); the identification numbers of the OTUs are indicated in the plots). (**n-o**) Tukey box plots indicating the relative abundance of the 8/36 OTUs in the mouse faecal microbiota (**n**) before (faeces_start) and (**o**) at the end of the four-week gavage period (faeces_end). Stars (*) indicate significantly different abundance for OTUs at the end of the gavage period compared to before (* p <0.05, ** p <0.01 and **** p <0.0001 according to Wilcoxon matched-pairs signed rank test). These results point to a lack of colonization in the conventional C57Bl/6J mice of key microbial taxa that were differentially abundant in the controls and cases human gut microbiota, and especially of taxa that were enriched in the cases gut microbiota.

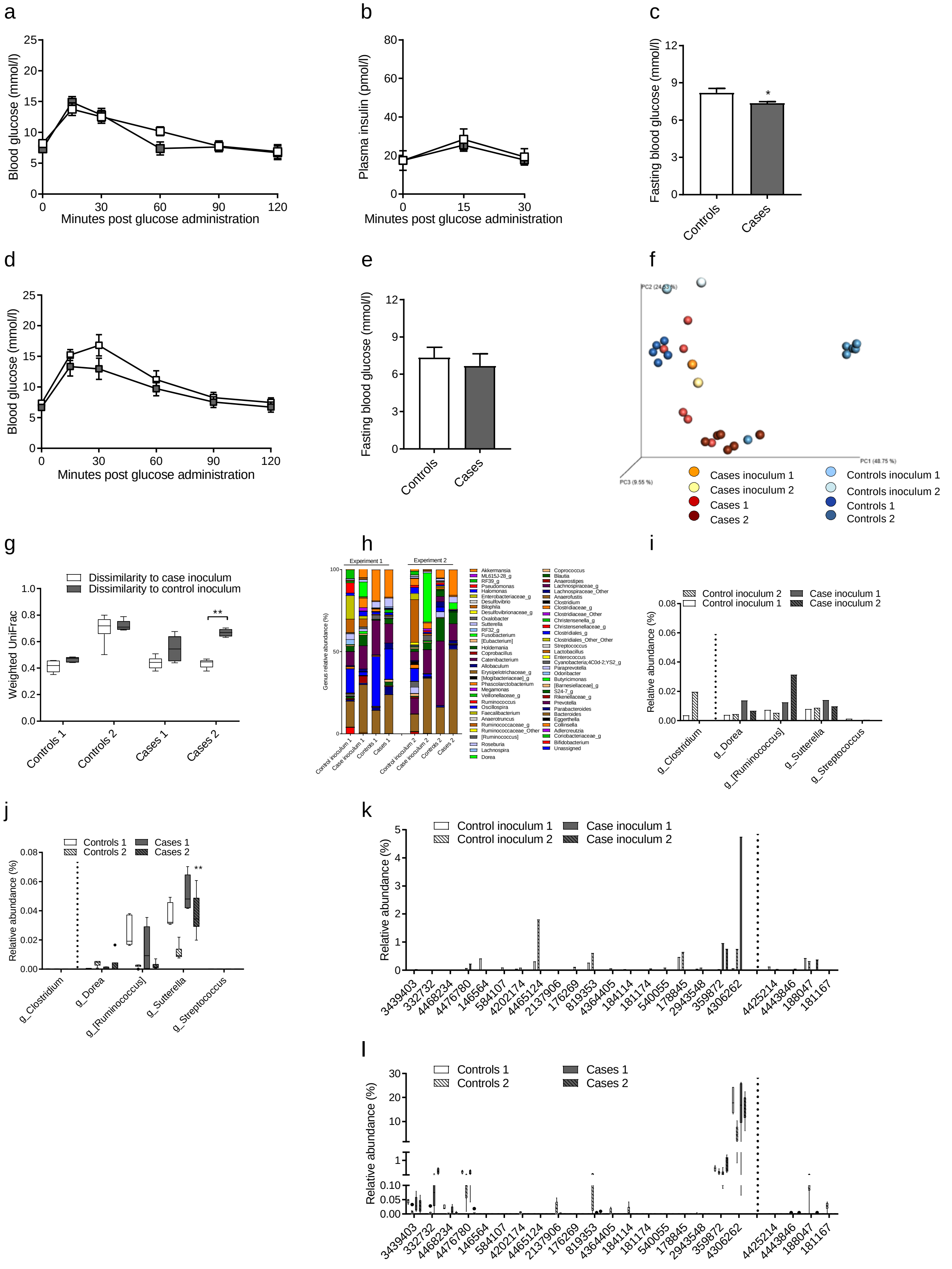
a
Blood glucose (mmol/l)
Minutes post glucose administration
b
Plasma insulin (pmol/l)
Minutes post glucose administration
c
Fasting blood glucose (mmol/l)
Controls
Cases
*
d
Blood glucose (mmol/l)
Minutes post glucose administration
e
Fasting blood glucose (mmol/l)
Controls
Cases
f
Cases inoculum 1
Cases inoculum 2
Cases 1
Cases 2
Controls inoculum 1
Controls inoculum 2
Controls 1
Controls 2
g
Dissimilarity to case inoculum
Dissimilarity to control inoculum
Weighted UniFrac
**
Controls 1
Controls 2
Cases 1
Cases 2
h
Experiment 1
Experiment 2
i
Control inoculum 2
Control inoculum 1
Case inoculum 1
Case inoculum 2
Relative abundance (%)
g_Clostridium
g_Dorea
g_[Ruminococcus]
g_Sutterella
g_Streptococcus
j
Controls 1
Controls 2
Cases 1
Cases 2
Relative abundance (%)
**
g_Clostridium
g_Dorea
g_[Ruminococcus]
g_Sutterella
g_Streptococcus
k
Control inoculum 1
Control inoculum 2
Case inoculum 1
Case inoculum 2
Relative abundance (%)
3439403
332732
4468234
4476780
146564
584107
4202174
4465124
2137906
176269
819353
4364405
184114
181174
540055
178845
2943548
359872
4306262
4425214
4443846
188047
181167
l
Controls 1
Controls 2
Cases 1
Cases 2
Relative abundance (%)
3439403
332732
4468234
4476780
146564
584107
4202174
4465124
2137906
176269
819353
4364405
184114
181174
540055
178845
2943548
359872
4306262
4425214
4443846
188047
181167

ESM Fig. 4 Faecal transfer from human donors with distinct glucose regulation in germfree Swiss Webster mice. (**a**) Glucose tolerance test of germfree Swiss Webster mice colonized with faecal microbiota from a screen detected diabetic donor (cases 1, n = 6) and a BMI matched control donor (controls 1, n = 5) (colonization 1); (**b**) Glucose stimulated insulin secretion during glucose tolerance test on mice from a; (**c**) Fasting blood glucose derived immediately before the glucose tolerance test described in a. (**d**) Glucose tolerance test of germfree Swiss Webster mice colonized with faecal microbiota from a screen detected diabetic donor (cases 2, n = 6) and a BMI-discrepant control donor (controls 2, n = 6) (colonization 2); (**e**) Fasting blood glucose derived immediately before the glucose tolerance test described in d. Mice were fed regular chow diet and glucose tolerance tests were performed two weeks after colonization. Data are presented as mean ± SE and statistical evaluation was performed by two-way ANOVA, repeated measurements with Bonferroni post hoc test (a-b and d) and Wilcoxon rank-sum tests (c and e). * $p < 0.05$ between controls and cases. White colour: controls. Grey colour: cases.

(**f**) Principal coordinate analysis (PCoA) plot of faecal microbiota composition in the Swiss Webster colonized mice based on weighted UniFrac. The dots in the PCoA plot indicate caecal samples collected at the termination of the experiment; the values in brackets on the axes show the percentage of variation explained by the first three principal coordinates (PC). We observed clustering of samples according to both colonization experiment (colonization 1 vs 2; $p = 0.001$, $r^2 = 0.286$; Adonis 999 permutations) and inoculum (cases vs controls inoculum; $p = 0.001$, $r^2 = 0.302$; Adonis 999 permutations). (**g**) Tukey box plots showing weighted UniFrac distances calculated to measure the dissimilarity of overall microbiota composition between mouse caecal samples and inoculum (0=identical; 1=completely different). The results show that controls from colonization 2 were highly and equally different from both the control and case inoculum, while controls from colonization 1 tended to be more similar to the case inoculum. This observation could explain why the diabetic phenotype of the donors did not precipitate in the recipient mice (** $p< 0.001$ according to Wilcoxon rank-sum test). (**h**) Bar plots showing the mean relative abundance of all microbial genera (n=65) identified in the inocula and in the caecal samples of the recipient mice. (**i**) Bar plots showing the relative abundance in the cases and control inocula of the five microbial genera that differed significantly in the human cohort. (**j**) Tukey box plots indicating the relative abundance of the five genera in the mouse caecal samples at the end of the two-week colonization period (** $p < 0.001$ between controls and cases in the same colonization experiment and according to Wilcoxon rank-sum test). (**k**) Bar plots showing the relative abundance in the cases and control inocula of the microbial OTUs that differed significantly in the human cohort; 23 out of the 36 OTUs that differed significantly in the human cohort were found among the highly abundant OTUs present in the inocula and in the mouse samples (19 OTUs, on the left of the dotted line, had increased abundance in the controls in the human cohort while 4 OTUs, on the right of the dotted line, had increased abundance in the cases in the human cohort (ESM Table 3); the identification numbers of the OTUs are indicated in the plots). (**l**) Tukey box plots showing the relative abundance of the 23/36 OTUs that differed significantly in the human cohort and that were found in the inocula and in the mouse caecal samples. Statistical evaluation was performed by Wilcoxon rank-sum test; *p* values for the comparisons of relative abundances between controls and cases in the same colonization experiment are listed in ESM Table 6. These results show that only part of the key microbial taxa were similarly differentially abundant in the recipient germfree Swiss Webster mice and in the human cohort (ESM Table 6).
